# Treatment and outcomes of crisis resolution teams: a prospective multicentre study

**DOI:** 10.1186/1471-244X-11-183

**Published:** 2011-11-22

**Authors:** Nina Hasselberg, Rolf W Gråwe, Sonia Johnson, Torleif Ruud

**Affiliations:** 1 Department of Research and Development at the Division Mental Health Services, Akershus University Hospital, Lørenskog, Norway; 2 Institute of Clinical Medicine, University of Oslo, Oslo, Norway; 3 Department of Research and Development at the Alcohol and Drug Treatment Health Trust in Central Norway, Trondheim, Norway; 4 Norwegian Centre for Addiction Research, Institute of Clinical Medicine, University of Oslo, Oslo, Norway; 5 Department of Mental Health Sciences, University College London, London, UK

## Abstract

**Background:**

Crisis resolution teams (CRTs) aim to help patients in acute mental health crises without admitting them to hospital. The aims of this study were to investigate content of treatment, service practice, and outcomes of crises of CRTs in Norway.

**Methods:**

The study had a multicentre prospective design, examining routine data for 680 patients and 62 staff members of eight CRTs. The clinical staff collected data on the demographic, clinical, and content of treatment variables. The service practices of the staff were assessed on the Community Program Practice Scale. Information on each CRT was recorded by the team leaders. The outcomes of crises were measured by the changes in Global Assessment of Functioning scale scores and the total scores on the Health of the Nation Outcome Scales between admission and discharge. Regression analysis was used to predict favourable outcomes.

**Results:**

The mean length of treatment was 19 days for the total sample (N = 680) and 29 days for the 455 patients with more than one consultation; 7.4% of the patients had had more than twice-weekly consultations with any member of the clinical staff of the CRTs. A doctor or psychologist participated in 55.5% of the treatment episodes. The CRTs collaborated with other mental health services in 71.5% of cases and with families/networks in 51.5% of cases. The overall outcomes of the crises were positive, with a small to medium effect size. Patients with depression received the longest treatments and showed most improvement of crisis. Patients with psychotic symptoms and substance abuse problems received the shortest treatments, showed least improvement, and were most often referred to other parts of the mental health services. Length of treatment, being male and single, and a team focus on out-of-office contact were predictors of favourable outcomes of crises in the adjusted model.

**Conclusions:**

Our study indicates that, compared with the UK, the Norwegian CRTs provided less intensive and less out-of-office care. The Norwegian CRTs worked more with depression and suicidal crises than with psychoses. To be an alternative to hospital admission, the Norwegian CRTs need to intensify their treatment and meet more patients outside the office.

## Background

The crisis resolution team (CRT) model of treating acute mental health crises outside in-patient wards has been implemented in some Western countries in the past decade [1,2]. With the adoption of CRTs in several Western countries in the past decade and in the UK and Norway, the implementation is part of national policies, it is important to evaluate the outcomes of crises after CRT care in ordinary clinical settings [3].

Guidelines or recommendations have been developed for the implementation of CRTs [4-6]. The teams should offer rapid assessment, intensive short-term home treatment, specialist multidisciplinary team interventions, reduced use of coercion, collaboration with the wider mental health care system and families/networks, and have gate-keeping functions for acute wards to a greater extent than outpatient clinics or in-patient wards. These key features of the CRT model are more a framework for delivering care and treatment than a specific type of treatment or therapy [1].

Recent studies in a range of UK settings, with both randomized and non-randomized designs, have suggested that CRT care is associated with a reduction in admissions to in-patient wards [7-13]. There is also some evidence that service users are more satisfied with CRT care than with standard care, although better study designs and response rates are required to be confident of this [1,7-16]. CRTs also seem to reduce care costs [17-19].

Apart from these findings, there is currently no clear evidence of any further clinical or social benefits of CRT care compared with standard care. In a Cochrane review, none of the studies found any differences in symptom outcomes, although none exclusively investigated crisis intervention, and the studies mainly ranged from the 1960s to the 1980s [19]. In the randomized controlled trial of CRT and standard care by Johnson et al., they found that symptoms, quality of life, social functioning, and adverse incidents, such as violence and self-harm, were similar between CRT and standard care after six months follow-up [8]. Another quasi-experimental study found no clear differences in symptoms, social functioning, or quality of life before and after the introduction of a CRT [9]. Barker et al. reported that carers said that the patients got better after CRT input, but that study had a low response rate (29%) [13].

Nor have most studies attributed any disadvantages to CRT care. The Cochrane review showed that treatment by a CRT was as safe as standard hospital care in terms of suicide, that home care reduced the family burden, and that there was no difference in the incidence of death [19]. Keown et al. reported that the number of suicides remained constant [11]. Bookle and Webber found that people of black ethnic origin used home treatments to the same extent as other ethnic groups in mental health crises [20]. However, Kingsford and Webber found that people from more socially deprived areas, older people, and those referred by enhanced community mental health teams had poorer outcomes after a CRT intervention [21]. In terms of admissions under the Mental Health Act in the UK, Keown et al. found that detentions under sections 2 and 3 of the Mental Health Act 1983 increased, whereas those under sections 5(2) and 5(4) declined following the introduction of crisis resolution and assertive outreach teams [11]. Barker et al. found a reduction in admissions under the Mental Health Act 1983 after CRTs began operating in Edinburgh [13]. These discrepancies indicate the need for further studies of the impact of CRTs on Mental Health Act admissions and on socially deprived people before we can draw any clear conclusions.

In an implementation study of the crisis resolution team model in Norway, it was found that the CRT model has been implemented without a rapid response, gate-keeping function and 24/7 availability [22].

The aim of the present study was to investigate and compare patients and CRTs with respect to: 1) content of treatment and service practices; 2) outcomes of crises; 3) predictors of favourable outcomes; and 4) where possible, compare Norwegian data with data from the UK.

## Methods

### Study design

This study had a naturalistic prospective pre-post multicentre design. The study was part of the Multicentre Study on Acute Psychiatry (MAP) in Norway. The multicentre study was planned and implemented by a national network to evaluate acute psychiatric services.

### Setting

Norway has a total population of 4.9 million people. The country is characterized by more rural areas and a lower population density than many other countries. The standard of living is generally high. Mental health service provision for adults consists of primary care and specialist mental health services. The primary health care services run by the 430 municipalities consist of general practitioners (GPs) and primary care mental health teams, usually staffed by psychiatric nurses, social workers, and occupational therapists. Many municipalities have residential services, day centres for people with mental health problems, and ambulatory care. The specialized mental health services run by 20 health authorities include 75 community mental health centres (CMHCs), hospitals with acute psychiatric wards and some specialized wards, and psychiatrists/psychologists in private practice. The CMHCs usually consist of outpatient clinics, in-patient wards, day care, and one or more specialized teams (case management teams, early intervention teams for first-episode psychoses, CRTs, and assertive community treatment teams). Specialized services for substance abuse are usually organized as part of the specialized mental health services in the health authorities.

In 2005, the national health authorities of Norway decided to implement the CRT model at all CMHCs, inspired by the implementation of CRTs in the UK. Establishing CRTs was given national policy priority, to improve the accessibility to specialized mental health services of people in mental health crisis and to offer these patients a rapid, intensive, and ambulatory alternative intervention to admission to an acute psychiatric ward. In a telephone survey of CRTs in Norway, 51 of the 76 CMHCs had established a CRT by 2010. Thirty of these only operated during office hours and one had 24/7 availability. When asked about their collaboration with families, 38 replied that they did collaborate and 31 replied that they most frequently met the patients at home. This indicates that the way the CRTs are organized and operate has not changed significantly since our data collection in 2005-2006, and that our data are still representative of these teams, although there are some indications of somewhat more home treatments in 2010 than in 2005-2006 [23].

In 2005, there were nine CRTs for adults in Norway, and eight of these teams participated in this study. The last CRT did not participate because it was undertaking a study of its own [24]. The target group of the CRTs was intended to be patients with mental health problems so severe and acute that without the involvement of a CRT, acute admission would usually be necessary [5]. The CRTs in this study were from all parts of Norway, varying from urban to rural areas, with catchment areas ranging from 65,000 to 115,000 inhabitants. They consisted of 4-19 team members, and the teams were multidisciplinary (mainly psychiatrists, psychologists, psychiatric nurses, and social workers). Three had a psychiatrist and six had a psychologist as a full-time member of the team. The intended response time was 12-48 hours and the intended length of treatment by these teams was between five consultations and eight weeks. The CRTs were similar in that they were not available 24/7, played no gate-keeping role for acute psychiatric wards, and treated patients who were not considered for hospital admission. There were variations between the CRTs in their opening hours, their authority to admit patients to acute in-patient wards, and their ability to facilitate early discharge from acute wards. The most usual referral routes to the CRTs were self-referral, and referral by GPs, CMHCs, primary care mental health teams, and casualty departments.

### Sample

In this multicentre study, the sample consisted of 680 patients and 62 staff members of eight CRTs. All patients referred during a three-month period, aged 18 years or more, and having face-to-face consultations with the CRTs were included in the study. There were no exclusion criteria.

Further patient and team characteristics have been presented in a previous paper [22].

### Data collection

The CRTs contributed to the planning of the study through their participation in semi-annual workshops in 2003-2005 in preparation for the study. The data were collected in 2005-2006. The CRTs included all patients referred during a three-month period, or longer if necessary to include 60 patients. The inclusion period started at different time points for different CRTs. The number of 60 patients was chosen to include a reasonable sample of patients from each team for a comparative data analysis. For patients seen for more than two months, the end of acute treatment was defined as being at two months, and the discharge assessment was performed at this point for these patients.

A registration form was designed to record information about the patients and the content of their treatments from admission to discharge. The form was piloted at two of the sites before its final revision. The data were collected by the clinicians in each CRT.

### Measures

At admission, socio-demographic characteristics and suicidal risk were assessed by the clinicians. Suicidal risk was coded as (i) no suicidal thoughts or plans, (ii) passive death wishes or suicidal thoughts without concrete plans, (iii) concrete suicidal plans or self-injury but no death intention, and (iv) self-injury and death intention. This suicidal scale was designed in collaboration with the National Centre for the Prevention of Suicide [25].

At discharge, a diagnosis according to the International Statistical Classification of Diseases and Related Health Problems, 10^th ^Revision (ICD-10) [26], the content of treatment, and the reason for discharge were recorded. The content of treatment included variables such as length of treatment, frequency of and participants in consultations, collaboration with other services, unwanted incidents, and pharmacological treatments.

Symptom severity and level of functioning were assessed at both admission and discharge using the Health of the Nation Outcome Scales (HoNOS) and Global Assessment of Functioning scale, split version (GAF) [27,28]. The patients who had one consultation were only rated once. The HoNOS consists of 12 subscales, each of which rates problems from 0 (no problem) to 4 (severe to very severe problem). In this study, the sums of scales 1-8 and 9-12 on HoNOS were calculated to give an overall measure of symptom severity and social problems, respectively. The subscales of HoNOS for overactive, aggressive, or disruptive behaviour, non-accidental self-injury, problems with drinking or drug-taking, problems with hallucinations and delusions, and problems with depressed mood were also included as the clinical scales most relevant to this study. The clinicians were trained in rating HoNOS in the half-day training seminar used in the UK, and all the clinicians had experience in rating GAF as a routine measure required for all treatment episodes in the mental health services. An earlier study, which used the same training for the clinicians, had shown acceptable inter-rater reliability (intra-class correlation coefficient [ICC] of 0.60-0.89) for the HoNOS subscales used in this paper [29].

The Community Program Practice Scale (CPPS) [30] was completed by each clinician. The CPPS is a questionnaire that measures practice and program climate of non-residential service models and consists of a 45-item scale on a five-point Likert scale (from 1 = strongly disagree to 5 = strongly agree) and with 13 subscales. For this study, the following six subscales were chosen as the most clinically relevant: case management, out-of-office contact, medication emphasis, team model, family orientation and involvement. The case management sub-scale measures whether the staff provide practical help to the patients, the out-of-office contact sub-scale measures to what degree the staff is working outside of the office, the medication emphasis sub-scale measures how much emphasis the team put on medication as a part of the treatment, the team model sub-scale measure whether more than one team member meet the patients, the family orientation sub-scale measures whether the team provide information or counselling for clients' family and the involvement sub-scale measures whether the staff members find their work interesting and challenging.

The HoNOS, GAF, and CPPS scales have shown satisfactory reliability and validity [30-32]. Several studies have indicated moderately high internal consistency and low item redundancy for the HoNOS sum score, and therefore support the instrument's use as a meaningful measure of symptom severity [31]. Söderberg found that when staff use patients' GAF scores to measure changes and outcomes, it might be necessary to use several raters for an individual patient for the GAF scales' reliability and validity to be satisfactory [33]. In this study, two or more raters filled in the registration form, including the GAF assessment score, for each patient.

A questionnaire completed by the team leaders assessed treatment approaches: response time, length of treatment, whether the CRT had a team approach with shared responsibility for the patient, collaboration with the wider mental health care system and families/networks, use of home treatment, and whether the CRT wanted to see the patient several times a week.

### Approval from authorities and contributions from user groups

The study was approved by the Regional Ethical Committee for Research in Health and by the Norwegian Data Inspectorate. The Directorate of Health and Social Affairs consented to the use of information from the health services. The data were collected from all patients without their written consent, because the Regional Ethical Committee for Research in Health had agreed to this insofar as it was important to include information on all patients. Representatives for the user organizations Mental Health Norway and the National Association of Relatives in Mental Health participated as a reference group and in the workshops to plan and prepare the study.

### Data analysis

HoNOS scales with missing values (average 5.5% across scales) were set to 0, because this was considered to be the most probable rating based on the skewed distribution with most patients rated 0, and on the assumption that clinicians most easily forgot to mark the rating when there was no indication of problems. This was also chosen in favour of imputation because it was the most conservative way to measure the severity of the patients' mental health problems. Diagnoses were missing for 53.5% and 17.4% of the patients in two teams and for 3.4%-10.4% in the other six teams. In Norway, only physicians/psychiatrists and psychologists are authorized to make ICD-10 diagnoses. The teams with the most missing values on the diagnosis variable operated without a physician/psychiatrist or psychologist as a regular member of the team and with nurses and social workers constituting the majority of their staff. In these teams, diagnoses were made by physicians who were not a part of the team. For this reason, the HoNOS scales were used instead of diagnoses in the analysis of the type and severity of the psychiatric problem.

One of the CRTs did not register the length of treatments (n = 46). An imputation of missing values was performed with a regression model. We identified the socio-demographic and clinical variables that predicted length of treatment. For each of these patients, we calculated the length of treatment based on the estimated coefficients of these predictor variables.

Descriptive and test statistics were assessed on all baseline variables according to whether the variables were categorical or continuous. Variations between the CRTs were also computed. In the analysis of treatment outcomes, we included only those patients who had received more than one consultation (n = 455). A paired-samples *t *test was used to evaluate the impact of the CRT interventions on the patients' clinical conditions by comparing the means of the pre-post test scores for the HoNOS total scores and the GAF scales. The calculation of the effect sizes was based on Cohen's *d*, defined as the difference between two means (pre- and post-treatment) divided by the standard deviation at admission [34].

A multilevel regression analysis was performed with the difference score for GAF symptoms as the dependent outcome variable. The ICC was 2.75% (ICC multiplied by 100), which indicated that the team level only contributed slightly to the explained variance. For this reason, a linear regression analysis was performed, with a stepwise backwards variable selection procedure. Potential predictors of a favourable outcome were chosen based on the guidelines for the implementation of CRTs both in relation to the target group and in clinical practice. The predictor variables selected were age, sex, being single, current employment, HoNOS scales 1-3 and 6-7 at admission, previous contact with mental health services, self-referral, length of treatment, intensity of consultations, doctor/psychologist participation in the consultations, collaboration with other mental health services and families/networks, pharmacological treatment, and the six selected subscales of the CPPS. The CPPS variables were used as team-level variables. Pairwise interaction tests were performed on all significant predictors.

SPSS software version 15 for Windows (SPSS Inc., Chicago, IL) was used for most of the data analysis. Multilevel regression analysis was performed using the software SAS 9.2. A significance level of 0.05 was used.

## Results

As shown in table 1, the 680 patients had a mean age of 40 years, 60% were female, 60% were single, and 25% were employed. The median number of patients per team was 80 (range, 46-147). The clinicians assessed patients to be at risk of suicide in about 60% of cases, and the mean GAF scores were 48.4 on the symptom scale and 49.6 on the functioning scale. The clinicians at the CRTs (n = 62) characterized themselves as focusing most often on involvement and least often on out-of-office contact. The analysis of the CRTs showed significant differences in the patients' characteristics and the service practices of staff members on most variables.

**Table 1:** Characteristics of 680 patients, 62 staff members, and eight CRTs

Variables	Total sample	Significance of the differences between teams
**Patient characteristics**		
Age (years), mean (SD)	40.1 (15.1)	0.066
Sex: n (%) female	396 (58.8)	0.507
Living alone: n (%)	396 (58.2)	<0.001**
Currently employed: n (%)	175 (25.7)	0.006**
Previous mental health service contact: n (%)	401 (59.0)	<0.001**
GAF mean (SD): Symptoms	48.4 (11.6)	<0.001**
Functioning	49.6 (12.6)	<0.001**
HoNOS mean (SD): Total	12.5 (6.26)	<0.001**
Total symptom severity (HoNOS 1-8)	7.6 (3.72)	<0.001**
Suicidal: n (%)		
No suicidal thoughts/plans	260 (39.8)	<0.001**
Passive death wishes/suicidal thoughts, no concrete plans	261 (39.9)	
Concrete suicidal plans/self-injury, but no death intentions	110 (16.8)	
Self-injury/death intentions	23 (3.5)	
**Service practices (CPPS) of the staff members**		
Case management: median (Q1-Q 3)	3.42 (3.05-3.63)	0.014**
Out of office contact: median (Q1-Q 3)	3.11 (2.89-3.72)	0.001**
Medication emphasis: median (Q1-Q 3)	3.34 (2.96-3.53)	0.033**
Team model: median (Q1-Q 3)	3.57 (3.02-4.17)	0.001**
Family orientation: median (Q1-Q 3)	3.66 (3.48-3.88)	0.018**
Involvement: median (Q1-Q 3)	4.44 (4.13-4.57)	0.131
**Team characteristics**		
Number of team members: mean (range)	9.1 (4.3-19.2)	
Individual staff member case-loads: mean (range)	2.5 (1.1-4.8)	
24/7 and gate-keeping function	None	
Team operates extended hours: n (%)	4 (50)	
Number of teams with specialist as a full-time part of the team: n (%)	4 (50)	

*p values from χ^2 ^tests, ANOVA (analysis of variance), and Kruskal-Wallis test; **significantly different

### Content of treatment

As shown in table 2, the mean length of treatment for the total sample was 19 days (SD = 24.4, range 0-97 days). Two hundred and twenty-five patients had a single consultation for CRT care/assessment, and the remaining 455 received treatment with a mean length of 29 days. We found no significant difference between these two groups in the severity of their mental health illnesses. The mean length of treatment differed significantly between the CRTs (range 7-30 days). Patients with depressive problems received significantly longer periods of treatment (21 days, SD = 22) than those with psychosis (13 days, SD = 21) or substance abuse (16 days, SD = 22). Patients with psychosis or substance abuse problems were frequently referred to other parts of the mental health service (about 10% were not), most often to GPs, psychiatric teams in primary care, CMHCs, or in-patient wards. The same applied to patients who received a single consultation (about 5% were not further referred).

**Table 2:** Contents of treatment

**Length of treatment:**		
All patients: days (SD) range	19.5 (24.4) 0-97	<0.001**
More than one consultation by a CRT: days (SD)	29.3 (24.8)	0.001**
**Frequency of consultations and co-operation:**		
One consultation by a CRT: n (%)	225 (33.1)	<0.001**
Consultations more than twice a week: n (%)	50 (7.4)	<0.001**
Doctor/psychologist participated in consultations	375 (55.1)	<0.001**
Inclusion of family/networks (consultations, meetings, other kinds of contact):	350 (51.5)	<0.001**
**Collaboration with other mental health services (consultations, meetings, or other kind of contact): n (%)**	486 (71.5)	0.038**
GPs	351 (51.6)	<0.001**
Community mental health centres	219 (32.2)	<0.001**
Psychiatric nurse/other professions in the municipality:	173 (25.4)	<0.001**
Acute in-patient wards	144 (21.2)	<0.001**
**Unwanted incidents: n (%)**		
Suicide attempts	14 (2.1)	
Self-harm	32 (4.7)	
Physical attacks on others	16 (2.4)	
Exposed to physical attacks from others	5 (0.7)	
**Pharmacological treatment: n (%)**		
Medication at the end of treatment	316 (42.0)	<0.001**
Antipsychotic medication: n (%)	138 (20.9)	
Antidepressant medication: n (%)	181 (26.6)	
Mood-stabilizing medication: n (%)	72 (10.6)	
Anxiety medication: n (%)	83 (12.2)	
Sleep medication: n (%)	91 (13.4)	
Other kind of medication: n (%)	5	
**Reasons for concluding treatment: n (%)**		
Concluded as planned	511 (75.1)	0.002**
Concluded earlier than planned	132 (19.4)	0.016**
Concluded later than planned	35 (5.1)	0.008**
**Other characteristics:**		
Individual care plan: n (%)	68 (10.0)	<0.001**
Use of coercion: n (%)	8 (0.1)	
Brought to CRT by the police: n (%)	27 (4.0)	

*p values from χ^2 ^tests, ANOVA, and Kruskal-Wallis test; "**significance of the difference between teams

In 7.4% of cases, the clinicians in the CRTs met the patient more than twice a week and the doctors and psychologists participated in 55% of the treatment episodes. The CRTs collaborated with other parts of the mental health services in 72% of cases and with families/networks in 52% of cases.

Pharmacological treatment was given to 42% of the patients. Few structured diagnostic interviews were used by the CRTs. Eight patients were under compulsory treatment.

With regard to the treatments, 75% of patients concluded them as planned.

### Outcomes of crises

Of the 455 patients who had more than one consultation, 262 had positive changes in the HoNOS total score and 256 in the GAF symptom score. As shown in table 3, the mean HoNOS total scores were 12.1 at admission and 10.02 at discharge. The corresponding figures for the GAF symptoms were 49.2 and 54.3, respectively. This indicates a significant improvement between admission and discharge, with the largest effect size on the GAF symptoms (*d *= -0.45). The effect sizes across the GAF and HoNOS total scores (*d *= 0.15-0.45) indicated a small or medium improvement after CRT care [34]. A comparison of the effect sizes of the CRTs showed that the effect sizes of the HoNOS and GAF total scores for the CRTs differed (*d *= 0.19-0.45).

**Table 3:** Treatment outcomes (n = 455): pre- and post-treatment data and effect sizes

	T1*		T2**				
	Mean	SD	Mean	SD	P	95% CI	d***
GAF symptoms	49.16	10.52	54.29	12.30	< 0.001	-5.87, -4.40	-0.45
GAF functioning	50.20	11.70	54.78	12.94	< 0.001	-5.32, -3.85	-0.37
HoNOS total	12.08	5.98	10.02	6.32	< 0.001	1.65, 2.47	0.34
HoNOS symptoms	7.25	3.52	5.72	3.63	< 0.001	1.24, 1.82	0.43
HoNOS sos probl	4.82	3.37	4.30	3.47	< 0.001	0.33, 0.73	0.15

Two-tailed

Results are presented as *t *values

* pre-treatment, ** post-treatment, ***effect size

Table 4 shows the numbers of patients with scores of ≥ 2 on the clinically relevant HoNOS subscales at admission and discharge. These scores decreased most on the depression scale (19.4) and least on the psychosis scale (3.0) and the substance abuse scale (2.7).

**Table 4:** Numbers of patients with scores of 2-4 on HoNOS subscales (n = 455)

	T1: n (%) score 2-4	T2: n (%) score 2-4	p
HoNOS 1 Overactive, aggressive, or disruptive behaviour	68 (14.9)	47 (10.3)	0.003
HoNOS 2 Non-accidental self-injury	80 (17.6)	39 (8.6)	<0.001
HoNOS 3 Problems with drinking or drug-taking	70 (15.4)	58 (12.7)	0.023
HoNOS 6 Problems with hallucinations or delusions	46 (10.2)	33 (7.2)	0.012
HoNOS 7 Problems with depressed mood	326 (71.7)	238 (52.3)	<0.001
HoNOS 9 Problems with relationships	212 (46.6)	172 (37.8)	<0.001

### Predictors of favourable outcomes of crises

Table 5 shows a linear multiple regression analysis of the significant predictors of favourable treatment outcomes, both unadjusted and adjusted for other variables. With adjustment for other variables, the length of treatment (p < 0.001), being male (p = 0.002), being single (p = 0.013), CRT focusing on out-of-office contact (p = 0.016), and having a problem with non-accidental self-injury (p = 0.017) were associated with a favourable outcome. A high degree of involvement of the team members (CPPS subscale) was negatively associated with outcome (p = 0.006). Current employment, having received consultations more than twice a week, and the participation of a doctor/psychologist in the consultations were significant predictive variables before we adjusted for other variables, but were not significant in the final multiple regression model.

**Table 5:** Predictors of favourable treatment outcomes

	Unadjusted*	p	95% CI	Adjusted**	p	95% CI
Age	-0.035	0.172	-0.085, 0.015	-0.038	0.147	-0.089, 0.013
Sex	-1.585	0.040***	-3095, -0.075	-2.499	0.002***	-4.069, -0.929
Single	1.282	0.096	0.230, 2.794	2.019	0.013***	0.436, 3.602
Non-accidental self-injury	0.718	0.027***	0.081, 1.355	0.820	0.017***	0.146, 1.494
Length of treatment	0.068	<0.001***	0.037, 0.099	0.068	<0.001***	0.037, 0.099
Out-of-office focus	0.708	0.038***	-0.879, 2.295	2.502	0.016***	0.476, 4.528
Involvement focus	-2.358	0.164	-5.681, 0.965	-5.770	0.006***	-9.843, -1.698
Currently employed	2.078	0.010	0.491, 3.667			
Consultations more than twice a week	3.481	0.005	1.058, 5.904			
Doctor/psychologist participated in consultations	1.474	0.050	-0.001, 2.950			
						
Interaction (sex × length of treatment)				0.029	0.001***	0.011, 0.046
Interaction (single × length of treatment)				0.038	<0.001***	0.019, 0.057
Interaction (non-accidental self-injury × length of treatment)				0.034	<0.001***	0.019, 0.050
Interaction (out-of-office focus × length of treatment)				0.020	<0.001***	0.011, 0.029
						
Explained variance				13.7%		

Multiple linear regression analysis of 455 patients and 62 staff members in eight CRTs

* Unstandardized bivariate regression coefficients

** Unstandardized multivariate regression coefficients

*** Significantly different (p < 0.05)

The pairwise interaction tests of all the significant predictors showed that a favourable outcome depended on the length of treatment: interaction effects p ≤ 0.001.

The regression model explained 13.7% of the variance.

## Discussion

The pattern of contact of the Norwegian CRTs was not characterized by intensive care, and there was an emphasis on depression and suicidal problems rather than on psychosis or substance abuse problems. The CRTs collaborated with other parts of the mental health system and with families/networks, but they had limited out-of-office and multidisciplinary contact.

### Content of treatment

Providing intensive home-based care is a key element of the CRT approach [1-4]. Half the CRTs in this study claimed to have focused on home treatment. Only one team claimed that they wanted to see patients several times a week, and only 7.4% of the patients had had more than twice-weekly consultations with any member of the clinical staff of the CRTs. A team focus on out-of-office contact was a predictor of a favourable outcome in the adjusted regression model. Compared with the UK, where home treatment programmes and frequent visits (usually at least daily) are considered key components of CRT care, the Norwegian treatment by CRTs can be characterized as short-term interventions with less intensive care, and with more outpatient care than home-based care. There might have been some changes related to home treatment since this study; the telephone survey mentioned in the setting section of this paper indicating more home treatments occurring in the Norwegian CRTs [23]. We suggest future studies should include measurement on actual home treatment frequency.

It has also been emphasized in this model that CRTs should be specialist multidisciplinary teams consisting of psychiatrists, psychologists, psychiatric nurses, social workers, and other social care professionals [1-4]. In this study, five of the CRTs lacked a full-time psychiatrist as part of the team. A national survey of CRTs in England also found a lack of full-time consultant psychiatrists (45% of teams had input from psychiatrist at a mean 0.5 full time) [35]. A significant proportion of the patients (about 45%) in our study did not meet a doctor or psychologist in a CRT during the treatment episode. This lack of consultant psychiatrists and psychologists is also reflected in the fact that many of the patients were not diagnosed by the CRTs during the treatment episode. In the unadjusted regression analysis, patients provided with a physician/psychologist during the consultations had better treatment outcomes. This lack of specialized professionals can restrict the CRTs' ability to provide comprehensive, multidisciplinary care.

A significant number of patients received only a single consultation for CRT assessment or care. Most of them were referred to other parts of the mental health services. This probably reflects the role of the CRTs as a kind of "triage" in the mental health system for patients with acute mental health problems. A key question is whether this screening process should be a function of outpatient clinics. The remaining group of patients received about four weeks of CRT care, with small to medium improvement. The size of the effect was not surprising given the brief period of the crisis intervention. Conversely, CRT care is a part a treatment chain in the mental health system. The clinical benefit of CRT care might be delayed, and may appear in another part of the mental health service.

We hypothesized that collaboration with other mental health services and families/networks would predict favourable outcomes, but it did not. In Norway, there has been particular emphasis on this part of CRT care. In the review of Winness et al. and the study of Hopkins and Niemiec of service users' experiences with CRTs, the inclusion of family members as part of the treatment and the staff's communication with other services were appreciated [15,16]. However, based on our study, we know little about the content of the contact with other parts of the mental health system or with families/networks, but only that there had been some form of contact (consultations, meetings, by phone, etc.).

### Outcomes of crises

This study indicates that patients may benefit from CRT care. However, patients with severe mental health illnesses were not common in our sample compared with studies in the UK. In studies of home-care acute psychiatric treatment based on data collected before the government proposed the establishment of nationwide CRTs in the UK, it was found that 53 - 62% of the patients had psychotic disorders [36-39]. In Johnson's two samples from 2005 37% and 40% had a psychotic disorder [8,9]. But the evidence is not wholly consistent; In a study of Barker et al from Edinburgh they found that 17% of the patients had psychotic symptoms [13]) and Tacchi found 13.5% with psychosis in a home treatment emergency response service in Newcastle [40]. With the lack of a randomized control group in this study, we cannot tell whether the patients would have progressed without CRT care (see the "Strengths and limitations" section below). The staff of these CRTs may also have overestimated the patients' improvement. Our measurement of the outcomes of crises was not based on patients' reports, but on the clinical staff's evaluations. By having the clinicians from the CRTs collect the data there is a risk of observer bias, especially with respect to rate HoNOS and GAF scales at initial assessment and discharge. Staff members from these teams were participating in the development of a new service in Norway, catering for people experiencing a mental health crisis. This might have increased the enthusiasm of the staff for their work, which may again have caused the staff to rate the patients' conditions better than they really were.

Patients with depressive symptoms showed the best outcomes from their crises, and non-accidental self-injury was also related to favourable outcomes. Patients with psychotic symptoms received shorter treatments, showed less improvement, and were most frequently referred to other parts of the mental health services. Our study indicates that because of the way in which Norwegian CRTs operate, they predominantly reach patients with depression and at risk of suicide.

The length of treatment was a highly significant predictor of favourable outcomes of crises, and an interaction effect showed that favourable treatment outcomes depended on the length of treatment. Although the interventions of the CRTs are meant to be brief, this finding indicates that these teams should provide intensive treatments for patients experiencing acute mental health crises rather than referring them to other parts of the mental health system or for rapid discharge. Then again, this finding may also indicate that people improve with time, regardless of any CRT care (see the "Strengths and limitations" section below).

In addition to the length of treatment, a team focus on out-of-office contact and suicidal problems, being male, and being single predicted favourable outcomes in the adjusted model. There were no significant differences between the sexes in the total severity of their symptoms or their social problems. The impact of CRT care may be greater for patients with little support from a social network.

The regression model in this study explained only a small part of the variance (13.7%). Despite the statistically significant results for several independent variables, it is clear that other unknown variables influenced the outcomes of these crises. CRT care is a complex intervention involving many factors. Given the variations in clinical practice and the significant variations in the social and clinical functioning of the patients in this study, it was likely that we would be unable to identify all the critical components required for favourable outcomes of these crises. The possible random distribution attributed to the unreliability of the GAF scale may also have reduced the amount of variance explained [33].

There were differences between the CRTs in the lengths of treatment and the outcomes of crises, insofar as the CRTs with best staffing provided the longest treatment episodes and had the best outcomes. However, the resources of the local mental health services in the catchment areas of the CRTs may have been intermediate variables that varied between the CRTs.

The proportions of compulsory treatments were low in these CRTs, but this is probably attributable to the small proportions of patients with severe mental health illnesses.

It is hard to interpret the finding that a high degree of involvement by the team members was negatively associated with the treatment outcomes. This might be a random finding. In contrast, this sub-scale measures whether the staff members find their work interesting and challenging and whether they are involved in their work. The implementation of the CRT model is a new way of treating patients experiencing mental health crises. Most staff members at the CRTs were enthusiastic and devoted to this new way of working. In their meetings with patients, this enthusiasm may have led to their over-involvement and excessive zeal, which may have caused negative outcomes of treatment.

### Strengths and limitations

The major strength of our design was its good external validity, because all patients treated at the CRTs were included and the data were obtained in routine clinical services, with no exclusion criteria.

The lack of a control group and of randomization was the most important limitations. Randomized controlled trials (RCTs) are generally considered the gold standard evidence for treatment effectiveness in medicine, although it has been argued that the complexity of interventions and the many factors that may cause outcomes to vary between settings may limit the usefulness of RCTs in mental health services research [41]. Because our study was an uncontrolled naturalistic study, the positive outcomes of crises after CRT care may have resulted from factors other than the CRT intervention. The patients in this study were included because they were experiencing an acute mental health crisis. Their improvements may have been spontaneous recoveries or the natural fluctuations that often characterize mental health problems.

## Conclusions

Our study shows that Norwegian CRTs provide less intensive and less out-of-office contact than UK CRTs, and they concentrate on depression and suicidal crises rather than psychoses. In the future implementation of CRT care in Norway, there should be an emphasis on improving the intensity of contact and ambulatory work, and an expansion of the target patients to include psychotic patients.

## Competing interests

The authors declare that they have no competing interests.

## Authors' contributions

TR, RWG, and NH designed the study and formulated the research questions. NH conducted the literature search. NH performed the statistical analysis and interpreted the data, with significant support from SJ and TR. The manuscript was written by NH and substantially revised by TR and SJ. The final version of the manuscript was prepared and revised by all authors. TR was the head supervisor of this manuscript and the project leader of the Multicentre Study of Acute Psychiatry in Norway (MAP).

## Pre-publication history

The pre-publication history for this paper can be accessed here:

http://www.biomedcentral.com/1471-244X/11/183/prepub
